# Clinician-identified problems and solutions for delayed diagnosis in primary care: a PRIORITIZE study

**DOI:** 10.1186/s12875-016-0530-z

**Published:** 2016-09-09

**Authors:** Lorainne Tudor Car, Nikolaos Papachristou, Adrian Bull, Azeem Majeed, Joseph Gallagher, Mona El-Khatib, Paul Aylin, Igor Rudan, Rifat Atun, Josip Car, Charles Vincent

**Affiliations:** 1 Department of Primary Care and Public Health, School of Public Health, Imperial College London, London, UK; 2 Imperial College Health Partners, London, UK; 3 gHealth Research Group, UCD Conway Institute, University College Dublin School of Medicine, Dublin, Ireland; 4 Centre for Global Health Research, Usher Institute of Population Health Sciences and Informatics, The University of Edinburgh Medical School, Edinburgh, UK; 5 Department of Global Health and Population, Harvard T H Chan School of Public Health, Boston, USA; 6 Department of Health Policy and Management, Harvard T H Chan School of Public Health, Boston, USA; 7 Health Services and Outcomes Research Programme, LKCMedicine, Nanyang Technological University, Singapore, Singapore; 8 Department of Experimental Psychology, Medical Sciences Division, University of Oxford, Oxford, UK

**Keywords:** Prioritization, Delayed diagnosis, Patient safety, Crowd-sourcing, Primary care, Clinicians

## Abstract

**Background:**

Delayed diagnosis in primary care is a common, harmful and costly patient safety incident. Its measurement and monitoring are underdeveloped and underutilised. We created and implemented a novel approach to identify problems leading to and solutions for delayed diagnosis in primary care.

**Methods:**

We developed a novel priority-setting method for patient safety problems and solutions called PRIORITIZE. We invited more than 500 NW London clinicians via an open-ended questionnaire to identify three main problems and solutions relating to delayed diagnosis in primary care. 113 clinicians submitted their suggestions which were thematically grouped and synthesized into a composite list of 33 distinct problems and 27 solutions. A random group of 75 clinicians from the initial cohort scored these and an overall ranking was derived. The agreement between the clinicians’ scores was presented using the Average Expert Agreement.

**Results:**

The top ranked problems were poor communication between secondary and primary care and the inverse care law, i.e. a mismatch between patients’ medical needs and healthcare supply. The highest ranked solutions included: a more rigorous system of communicating abnormal results of investigations to patients, direct hotlines to specialists for GPs to discuss patient problems and better training of primary care clinicians in relevant areas. A priority highlighted throughout the findings is a need to improve communication between clinicians as well as with patients. The highest ranked suggestions had the highest consensus between experts.

**Conclusions:**

The novel method we have developed is highly feasible, informative and scalable, and merits wider exploration with a view of becoming part of a routine pro-active and preventative system for patient safety assessment. Clinicians proposed a range of concrete suggestions with an emphasis on improving communication among clinicians and with patients and better GP training. In their view, delayed diagnosis can be largely prevented with interventions requiring relatively minor investment. Rankings of identified problems and solutions can serve as an aid to policy makers and commissioners of care in prioritization of scarce healthcare resources.

**Electronic supplementary material:**

The online version of this article (doi:10.1186/s12875-016-0530-z) contains supplementary material, which is available to authorized users.

## Background

Delayed diagnosis and other diagnostic errors are more common, costly and harmful than any other patient safety threat [1, 2]. While data on delayed diagnosis is lacking, diagnostic errors overall are the 6th leading cause of death in the United States [3, 4]. They affect most Americans at least once in their lives and contribute to 80,000 deaths annually [5]. Primary care is particularly liable to delayed diagnosis since a) it is where the majority of patient-doctor encounters happen; b) it encompasses a diverse and often complex range of conditions in all age groups; c) its role is to manage risk [6].

Internationally, leading organisations are calling for dramatic strengthening of the evidence base about the causes of and solutions to delayed diagnosis and other diagnostic errors [5, 7]. However, delayed diagnosis is difficult to measure and has so far mostly been considered an individual failure rather than an organizational or system problem [8]. There is a lack of consensus on the definition of diagnostic error reflecting the complexity of the diagnostic process. It is important to acknowledge and address this diversity in terminology to allow comparisons between studies and progress in this area of patient safety research [9]. The shortage of comprehensive diagnostic safety measurement tools is accompanied by frequent omission of delayed diagnosis from patient safety policies [10]. Research methods that have so far been used for the analyses of diagnostic errors include analysis of malpractice claims, autopsies, surveys, case reviews and incident reporting [11]. These methods focus at a selected sample of diagnostic errors and are backward-looking, i.e. they reveal harm that has already happen [12, 13]. Furthermore, it is important to note that reducing delayed diagnosis in contemporary medicine comes with a risk of overdiagnosis which can lead to severe harm due to unnecessary treatment or unnecessary diagnostic tests [14].

Healthcare staff views offer important insight into patient safety culture and can help in anticipating future harm. A recent study showed that staff feedback predicted organisational level of patients safety [15]. Yet, a recent review on whistleblowing in the NHS showed that healthcare providers who voice their safety concerns face appalling consequences [16]. Rather than waiting to learn from tragic events we need more routine assessments of staff views on safety priorities and potential interventions. In this study, using a novel approach, we invited clinicians to anonymously share their views on the causes of delayed diagnosis and on the interventions facilitating a timely and accurate diagnosis.

## Methods

We adopted a definition of delayed diagnosis as “a diagnosis that was unintentionally delayed while sufficient information was available earlier” [17].

We developed PRIORITIZE method, by modifying the Child Health and Nutrition Research Initiative (CHNRI) methodology for patient safety context to determine the main problems and solutions relating to delayed diagnosis in primary care [18–20]. The method utilises participants’ perspectives to customize a priority agenda based on the local context and needs. The CHNRI methodology has been used widely to inform policy makers, funders and international organizations about research gaps and resource priorities [20–22].

**Fig. 1:**
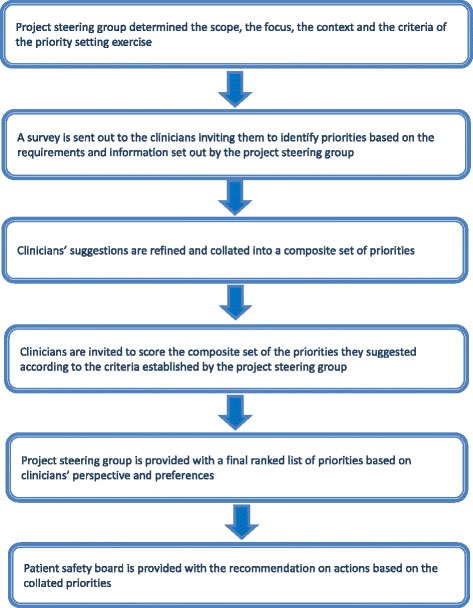
PRIORITIZE methodology flow diagram

The PRIORITIZE approach consisted of the following steps:

1. Project steering group determined the scope, the focus, the context and the criteria of the priority setting exercise
2. A survey was sent out to the clinicians inviting them to identify priorities based on the requirements and information set out by the project steering group
3. Clinicians’ suggestions were refined and collated into a composite set of priorities
4. Clinicians were invited to score the composite set of the priorities they suggested according to the criteria established by the project steering group
5. The project steering group was provided with a final ranked list of priorities based on clinicians’ perspective
6. Final ranked list of priorities guided the Patient Safety Board in shaping a list of actions and timeline for those as well as their wider dissemination back to clinicians and other stake-holders (Fig. 1).

While the CHNRI methodology invites experts in the relevant field to nominate research priorities, PRIORITIZE focused on priorities in healthcare services delivery and identified clinicians’ as experts. The PRIORITIZE methodology determined priorities by focusing on the topic from two complementary angles: problems and solutions. The final output of this approach is presenting the top priorities categorized according to the level at which these could be implemented: a) actions for general practitioners; b) actions for general practice organisations; and c) actions for health system custodians.

This study was deemed to be a service evaluation and quality and safety improvement initiative and consequently did not require ethics or research governance approval according to the UK’s Health Research Authority guidance [23]. During the study’s first stage, the project steering group (Imperial College Health Partners) considered previous evidence on patient safety in primary care in the UK and decided to focus on medication safety (presented elsewhere) and delayed diagnosis. They also chose the criteria guiding prioritisation of collated suggestions, i.e. scoring of problems and solutions (Table 1).

**Table 1:** Scoring criteria

Problems	Solutions
Frequency: This patient safety threat is common Severity: This patient safety threat leads to high rates of mortality, morbidity and incapacity Inequity: This patient safety threat affects lower socio-economic groups or ethnic minorities more than other groups Economic impact: The consequences of this patient safety threat are costly to the healthcare system Responsiveness to solution: This incident is amenable to a solution within 5 years	Feasibility: The implementation of this solution is feasible Cost-effectiveness: This solution is cost-effective Potential for saving lives: This solution would save lives

Following a review of relevant literature, we developed an open-ended questionnaire for clinicians to identify main problems and solutions relating to delayed diagnosis in primary care. The survey also collected data on the professional group of the participants. We piloted the questionnaire on a smaller sample of general practitioners and trainees and amended it according to collected feedback (Additional file 1). The questionnaire was delivered in a paper-based and an equivalent online version. The questionnaire consisted of seven questions and no definition of delayed diagnosis was provided. Study participants were asked to comment of delayed diagnosis in primary care in general. It was disseminated through email lists, snowballing (participants were asked to forward the survey to colleagues), and visits to general practices in north west (NW) London. We sampled academic and non-academic general practitioners, trainees, pharmacists and nurses.

We performed a content analysis on the collected ideas using open coding to categorise the free-text responses. Suggested ideas which were sufficiently similar were merged. In the second phase we asked clinicians to categorize the ideas using four options: 1 for ‘Yes - I agree with the statement’, 0 for ‘No - I do not agree with the statement’, 0.5 for ‘Unsure - I am unsure whether or not I agree’ and blank (no response) for ‘Unaware – I do not feel sufficiently familiar or confident to score this suggestion’ (Additional file 2). As the scoring was time demanding (an average 1 h to complete) we offered a token payment to the respondents in a form of a £100 grocery voucher. Clinicians who performed scoring of the priorities were randomly selected from the initial cohort of primary care clinicians. We ended the enrolment after collecting at least 50 completed sheets as per CHNRI methodology guidance (personal communication I. Rudan).

We computed the scores for the suggested priorities as the mean of the scores for each of the five criteria for problems ranging from 0 to 100. Because of the number of participants, the non-standardised categorical nature of our data together with us allowing missing responses, and finally the number of our different criteria, Kappa statistics were deemed to be an inappropriate test to calculate inter-rater agreement. Instead, we report the average expert agreement (AEA) [24]. AEA is the proportion of scorers who chose the mode (the most common score) for each research question. Although AEA does not give an indication of statistical significance of difference between scorers, it is of relevance to policy makers as it provides an indication of the degree of agreement between clinicians. The AEA was calculated using the following formula:

$$ \mathrm{A}\mathrm{E}\mathrm{A} = \frac{1}{5}\times {\displaystyle \sum_{\mathrm{q}=1}^5\frac{\mathrm{N}\ \left(\mathrm{scorers}\ \mathrm{who}\ \mathrm{provided}\ \mathrm{the}\ \mathrm{most}\ \mathrm{frequent}\ \mathrm{response}\right)}{\mathrm{N}\ \left(\mathrm{scorers}\right)}} $$

$$ \mathrm{A}\mathrm{E}\mathrm{A} = \frac{1}{3}\times {\displaystyle \sum_{\mathrm{q}=1}^3\frac{\mathrm{N}\ \left(\mathrm{scorers}\ \mathrm{who}\ \mathrm{provided}\ \mathrm{the}\ \mathrm{most}\ \mathrm{frequent}\ \mathrm{response}\right)}{\mathrm{N}\ \left(\mathrm{scorers}\right)}} $$

(where q is a question that experts are being asked to evaluate competing patient safety threats (in this case diagnostic errors), ranging from 1 to 5 for problems and 1 to 3 for solutions).

In our analysis of the proposed problems and solutions, we used a framework in which causes of diagnostic errors are categorized into system, cognitive and patient-related factors [10, 17]. Further to that problems and solutions were also organised in terms of the point of diagnostic process they refer to: 1. Access and presentation, 2. Patient-practitioner clinical encounter, 3. Performance and/or interpretation of diagnostic tests, 4. Referral and consultation and 5. Follow-up and tracking of diagnostic information [25]. Solutions were categorized according to the type of organizational intervention for decreasing diagnostic errors, i.e. technique, personnel changes, educational interventions, structured process changes, technology-based intervention and additional review (Additional file 3) [26]. The assigned scores allowed ranking of solutions.

**Fig. 2:**
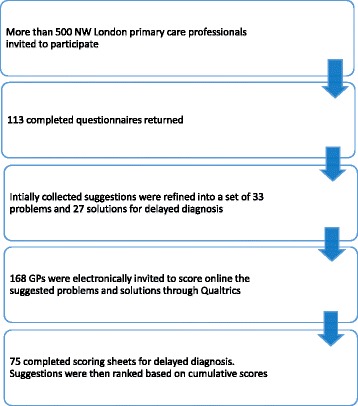
Participants flow diagram

In the first phase we invited > 500 primary care clinicians and received 113 completed questionnaires (response rate ~22.6 %) with the majority completed by GPs (*n* = 85, 75.2 %) (Additional file 4). They proposed 173 problems and 112 solutions relating to delayed diagnosis that were thematically merged into 33 distinct problems and 27 solutions. From the phase 1 cohort, 168 randomly selected GPs were invited to score the composite list of suggestions resulting in 66 fully completed scoring sheets (Fig. 2).

## Results

The top ranked problems leading to delayed diagnosis were poor communication between secondary and primary care and the inverse care law, i.e. the principle that the availability of good medical or social care tends to vary inversely with the need of the population served (Table 2). The highest ranked solutions to delayed diagnosis were development of a more rigorous system for communicating abnormal results to patients, direct hotlines to specialists to discuss patient problems and clear referral guidelines and pathways for common conditions (Table 3).

**Table 2:** Clinicians’ identified top ten problems leading to delayed diagnosis in primary care^a^

Rank	Problems leading to delayed diagnosis in primary care	Total priority score	Type of factor leading to diagnostic error	Breakdown points in the diagnostic process
1	Poor communication between secondary and primary care; e.g. investigations that are ordered by secondary care are not visible in primary care	78.2	System factor	Referral & consultation
=1	Inverse care law i.e. those who most need medical care are least likely to receive it. Conversely, those with least need of health care tend to use health services more and more effectively	78.2	System and patient-related factor	Access & presentation
3	Patients attending other services such as A&E walk-in centres instead of seeing their own GP	76.6	System and patient-related factor	Access & presentation
4	Multiple symptoms or co-morbidities masking the real problem	76.3	Cognitive factor	Patient-practitioner encounter
5	Lack of continuity of care - seeing different GPs’ for the same problem and never being able to follow ‘a case’ through properly	76.3	System factor	Patient-practitioner encounter
6	Time constraints such as the 10 min consultations that lead to incomplete history-taking and patient examination	76.3	System factor	Patient-practitioner encounter
7	Lack of patient awareness of ‘red flag’ symptoms	76	Patient-related factor	Access & presentation
8	Patient’s delay in presenting symptoms (e.g. “I have had blood in my urine for a year”)	75.4	Patient-related factor	Access & presentation
9	Psychiatric co-morbidity (the co-occurrence of two or more psychiatric diagnoses) leading doctors to insufficient attention to physical symptoms	74.5	Cognitive factor	Patient-practitioner encounter
10	Language and cultural barriers between the GP and the patient	73	System and patient-related factor	Patient-practitioner encounter

(Clinicians scored problems using the following criteria: frequency, severity, inequity, economic impact and responsiveness to solution (Table 1). The scoring options were 1 for “yes (e.g. this problem is common)”, 0 for “no (e.g. this problem is uncommon)”, 0.5 for “unsure (e.g. I am unsure if this problem is common)” and blank for “unaware e.g. I do not know if his problem is common)”. Total Priority score is the mean of the scores for each of the five criteria and is ranging from 0 to 100. Higher ranked problems received more “Yes” responses for each of the criteria and a higher score)

^a^All tables use clinicians’ verbatim statements which were only exceptionally reworded for clarity

**Table 3:** Clinicians’ identified top 10 solutions for delayed diagnosis in primary care

Rank	Suggestions for solutions to delayed diagnosis in primary care	Total priority score	Type of interventions to decrease delayed diagnosis	Breakdown points in the diagnostic process
1	To have more rigorous systems in place for communicating abnormal results to patients	92.3	Structured-process change	Follow-up
2	Direct hotlines to specialists to discuss patient problems	91.4	Structured-process change	Referral & consultation
3	Clear referral guidelines and pathways for other common conditions (not just cancer)	88.4	Structured-process change	Referral & consultation
4	Improve handovers	86.9	Structured-process change	Referral & consultation
6	To have “affordable” GP update courses	86.3	Educational intervention	Patient-practitioner encounter
5	Better training of GPs in spotting warning signs of serious conditions, diagnosis that are easily missed and safety netting	86.3	Educational intervention	Patient-practitioner encounter
7	Review of every delayed diagnosis to learn how, why and whether it could be prevented in the future	85.4	Additional review & education	NA
8	Better ways of informing patients that their results are ready and what the next best steps would be	84.8	Structured-process change	Follow-up
9	Training in decision making and reinforcing the concept on ongoing reflection to continuous consideration of differential diagnosis	84.5	Educational intervention	Patient-practitioner encounter
10	Have easier access to secondary care for the patients that GPs are worried about	83.9	Structured-process change	Referral & consultation

(Clinicians scored solutions using the following criteria: feasibility, cost-effectiveness and potential for saving lives (Table 1). The scoring options were 1 for “yes (e.g. this solution is feasible)”, 0 for “no (e.g. this solution is unfeasible)”, 0.5 for “unsure (e.g. I am unsure if this solution is feasible)” and blank for “unaware (e.g. I do not know if this solution is feasible)”. Total Priority score is the mean of the scores for each of the three criteria and is ranging from 0 to 100. Higher ranked solutions received more “Yes” responses for each of the criteria and a higher score)

Several proposed problems indirectly contribute to the inverse care law as their impact is more prominent in patients who are in greatest need, such as short consultations, presence of comorbidities, low health literacy and high GP stress (Additional file 5). The top ranked problems resulting in delayed diagnosis were mostly system and patient-related factors (Table 2).

Patient related factors addressed patients’ delayed presentation to care while system factors referred to poor communication between the ‘levels of care’, the short duration of the consultation and lack of care continuity (Additional file 5). Language and cultural barriers between the GP and the patient, lack of patient awareness of ‘red flag’ symptoms and patient delay in presenting with significant symptoms were identified as problems primarily affecting lower socio-economic groups and ethnic minorities (Additional file 5).

**Fig. 3:**
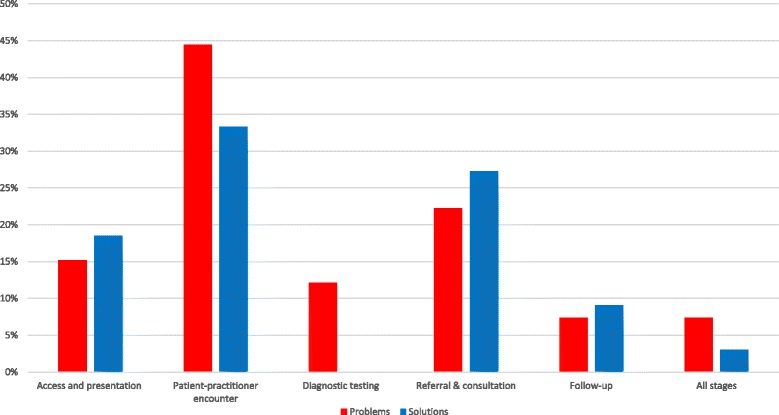
Comparison of problems and solutions related to delayed diagnosis in primary care in terms of the diagnostic process breakdown point

Difficulties in patients’ access and presentation to care were considered the most important problems leading to delayed diagnosis, while the highest ranked solutions mostly addressed improvements in communication with secondary care and training (Tables 2 and 3). Cancer was the only condition which was specifically mentioned among the proposed problems and solutions (Additional files 5 and 6). Overall, most of the proposed problems and suggestions revolved around patient-practitioner encounter (Fig. 3). The highest ranked suggestions had the highest AEA, i.e. there was a stronger consensus among the clinicians in regards to the top suggestions compared to those ranked lower. Proposed solutions received higher AEA scores compared to problems (Additional file 5).

## Discussion

Clinicians identified a wide range of problems leading to and solutions for delayed diagnosis in primary care. Poor communication between secondary and primary care and the inverse care law, i.e. a mismatch between patients’ medical needs and healthcare supply were considered the key problems leading to delayed diagnosis. Lack of continuity of GP care, late or inappropriate access to care and the presence of psychiatric and other comorbidities were all ranked among the top ten problems leading to delayed diagnosis.

Improving communication between clinicians and with patients was once again reaffirmed as one of the key overarching priorities for mitigating patient safety incidents and improving patient outcomes [27–29]. The term communication covers a multitude of different practices and activities and this study has highlighted the particular importance of communication of test results. Research shows that primary care physicians order laboratory tests in nearly a third of all patient encounters [30]. And of those, up to one-third of patients are not notified of abnormal test results although failure to communicate them can cause significant patient harm [30, 31].

The inverse care law’s contribution to delayed diagnosis is not surprising. Socioeconomically deprived patients are more likely to report difficulties obtaining needed health care [32, 33]. This is aggravated through short consultations, presence of comorbidities, higher GP stress and lower patient health literacy even within a universal health coverage system such as the NHS [34]. These contributing factors were identified and ranked highly as the most important threats to accurate and timely diagnosis in primary care patients overall. However, their impact is even more prominent in patients who need the care most which corroborates the inverse care law.

By inviting providers to nominate both problems and solutions, we gained a more complete insight into providers’ views on patient safety priorities. Although cognitive factors are thought to be the commonest contributory factors to diagnostic errors [17, 35], our respondents considered them less important compared to patient-related and system factors. One of the reasons behind this could be that study participants found it more difficult to recognize and report problems related to their personal responsibilities in diagnostic delays. Physicians’ surveys showed that physicians underappreciate the likelihood of diagnostic errors and reference system and patient factors when asked about cognitive errors [2]. A more complete picture emerges from the solutions proposed as clinicians considered educational interventions as essential for the improvement of diagnostic process.

A systematic review on diagnostic challenges in primary care shows that cognitive errors were more likely to occur when the patient was unfamiliar to the clinician, and had atypical presentations of common diseases or “distracting” comorbid conditions [30]. Clinicians in the UK consider fragmentation of care and poor continuity as a key reason behind delayed dancer diagnosis [36, 37]. Similarly, in our study, lack of continuity of GP care, late or inappropriate access to care and presence of psychiatric and other comorbidities were all ranked among top ten problems leading to delayed diagnosis.

It is gratifying that the proposed solutions, which focused mostly on process changes to improve referrals, quality of consultations and decision-making, and educational interventions aimed at improving diagnostic knowledge and skills have an existing (albeit weak) evidence base and improve outcomes [10, 26, 38]. Support of both theoretical and empiric evidence for these and other identified interventions in the NW London context, such as, asking for a second opinion and help from other clinicians, the use of decision support tools and electronic support systems to improve follow-up of abnormal test results should put them high on priority agenda for implementation [39].

### Strengths and limitations

PRIORITIZE has many strengths from its transparency and easy reproducibility, participation of a groupof doctors; anonymity, where worries, suggestions and ideas can be voiced in a frank and blame-free way – often expressing significant concerns or frustrations [40–42]. It offers a novel critical insight into patient safety from a ‘collective wisdom’ perspective rather than an analysis of patient safety incidents or (verbal) autopsies. It provides an insight not just where incidents happened but more importantly where the largest risks lie for them to happen again at a system level. It is founded on the concept of crowdsourcing and is particularly valuable for insights into topics such as patient safety which is still largely a taboo, emotionally laden, charged with guilt or risk of blame and avoided in discussions. Past surveys focused on diagnostic errors determined the main diagnostic process threats and solutions based on how frequently they occurred [43, 44]. We used other relevant and well-defied criteria such as severity, impact, costliness to the healthcare system and solvability of a problem. This priority-setting approach is based on the notion of scarcity and finite healthcare resources that can be invested in improvements of policies and practice.

A limitation of this study concerns generalizability and validity of the findings. The respondents were self-selected and potentially differed from the non-respondents. The study findings may not be generalizable to other healthcare settings (e.g. rural) or healthcare systems which are different to the UK. Nevertheless, they strongly resonate with the international literature in terms of what in general are features of safe primary care and good diagnostic processes and as such should form an important checklist for considerations beyond the study setting [17]. This methodology could be applied relatively easily to other groups and populations to expand our understanding of safety priorities and other issues. As a future step, we could also collect information from secondary care providers and patients too, to analyse consistency among the collated suggestions.

We believe that whilst our findings are significant, the method is at an early stage and would benefit from triangulation. The method could evolve and test whether, for example, providing examples to guide the specificity and type of the suggestions (e.g. error producing conditions vs adverse events), adding a longitudinal perspective, could give us further insight. Additional modes of information analysis are also an option e.g. determining the level at which the improvements need to be implemented, be it at the system, practice, individual level or a combination of them.

## Conclusions

Clinicians identified a wide range of valuable, concrete suggestions to prevent delayed diagnosis highlighting the need for improvement in communication among clinicians and with patients and better GP training. In their view, delayed diagnosis can be largely prevented with interventions requiring relatively minor investment. However, in current climate of limited and reducing resources implementing those interventions may be more challenging and competing priorities may prevent their adoption and implementation.

PRIORITIZE, a novel priority-setting approach, allows healthcare commissioners and policymakers to gather staff feedback, trigger their involvement, evaluate their views on patient safety priorities, assess organizational safety culture and ultimately align policies with the collated information. It also offers decision-makers an opportunity to define the scope and focus of the priority-setting exercise as well as the granularity of the responses. Rankings of identified problems and solutions in this approach can serve as an aid in prioritization of scarce healthcare resources.

PRIORITIZE also implements new policy direction in the UK of involving more healthcare staff in patient safety [45] and is complementary to current patient safety tools [46]. For clinicians PRIORITIZE is empowering and could provide a framework for staff calibration, i.e. comparison between the clinicians’ self-assessment and assessment of the healthcare system overall in terms of patient safety threats and actual errors. However, future studies and more evidence on the validity and reliability of this approach is needed.

PRIORITIZE is highly feasible, informative and scalable approach. We propose exploring whether it could be embedded into the mechanism of annual appraisal of staff as a routine pro-active and preventative systems to detect the vulnerabilities at different levels of care. As a system-wide initiative it could increase awareness of patient safety threats and improve organisational culture and attitudes. Central collection of this data could allow country-wide comparison and implementation of locally tailored-interventions.

## Additional files

Additional file 1: Initial questionnaire on problems and solutions related to medication safety and delayed diagnosis in primary care. (DOCX 65 kb) Additional file 2: Scoring questionnaire. (DOCX 85 kb) Additional file 3: Categories of Organizational Interventions to Decrease Diagnostic Errors. (DOCX 13 kb) Additional file 4: Characteristics of the respondents to the initial questionnaire. (DOCX 12 kb) Additional file 5: Ranking of all (33) problems leading to delayed diagnosis from primary care clinicians’ perspective (AEA range: 0 to 1). (DOCX 19 kb) Additional file 6: Ranking of all (27) solutions to delayed diagnosis from primary care clinicians’ perspective (AEA range: 0 to 1). (DOCX 17 kb)

## Abbreviations

AEA: Average experts’ agreement
GP: General practitioners
NW: North-west

## References

[1] Shekelle PG, Pronovost PJ, Wachter RM, Taylor SL, Dy SM, Foy R, Hempel S, McDonald KM, Ovretveit J, Rubenstein LV, Adams AS, Angood PB, Bates DW, Bickman L, Carayon P, Donaldson L, Duan N, Farley DO, Greenhalgh T, Haughom J, Lake ET, Lilford R, Lohr KN, Meyer GS, Miller MR, Neuhauser DV, Ryan G, Saint S, Shojania KG, Shortell SM (2011). Advancing the science of patient safety. Ann Intern Med.

[2] Berner ES, Graber ML (2008). Overconfidence as a cause of diagnostic error in medicine. Am J Med.

[3] Graber ML (2013). The incidence of diagnostic error in medicine. BMJ Qual Saf.

[4] Leape LL, Berwick DMBD (2002). Counting deaths due to medical errors [letter]. JAMA.

[5] National Academies of Sciences, Engineering AM (2015). Improving diagnosis in health care.

[6] Bishop TF, Ryan AM, Ryan AK, Casalino LP (2011). Paid malpractice claims for adverse events in inpatient and outpatient settings. JAMA.

[7] National Patient Safety Agency. Delayed diagnosis of cancer: thematic review. London: National Reporting and Learning Service; 2010.

[8] Wachter RM (2010). Why diagnostic errors don’t get any respect—and what can be done about them. Health Aff.

[9] Zwaan L, Singh H (2015). The challenges in defining and measuring diagnostic error. Diagnosis.

[10] Singh H, Graber ML, Kissam SM, Sorensen AV, Lenfestey NF, Tant EM, Henriksen K, LaBresh KA (2012). System-related interventions to reduce diagnostic errors: a narrative review. BMJ Qual Saf.

[11] Zwaan L, Schiff GD, Singh H (2013). Advancing the research agenda for diagnostic error reduction. BMJ Qual Saf.

[12] Singh H, Sittig DF (2015). Advancing the science of measurement of diagnostic errors in healthcare: the Safer Dx framework. BMJ Qual Saf.

[13] Lawton R, McEachan RRC, Giles SJ, Sirriyeh R, Watt IS, Wright J (2012). Development of an evidence-based framework of factors contributing to patient safety incidents in hospital settings: a systematic review. BMJ Qual Saf.

[14] Hoffman JR, Kanzaria HK (2014). Intolerance of error and culture of blame drive medical excess. BMJ.

[15] Lawton R, O’Hara JK, Sheard L, Reynolds C, Cocks K, Armitage G, Wright J (2015). Can staff and patient perspectives on hospital safety predict harm-free care? An analysis of staff and patient survey data and routinely collected outcomes. BMJ Qual Saf.

[16] Francis R. Freedom to speak up. London, UK: 2015.

[17] Graber ML, Franklin N, Gordon R (2005). Diagnostic error in internal medicine. Arch Intern Med.

[18] Rudan I, Chopra M, Kapiriri L, Gibson J, Ann Lansang M, Carneiro I, Ameratunga S, Tsai AC, Chan KY, Tomlinson M, Hess SY, Campbell H, El Arifeen S, Black RE (2008). Setting priorities in global child health research investments: universal challenges and conceptual framework. Croat Med J.

[19] Rudan I, Gibson JL, Ameratunga S, El Arifeen S, Bhutta ZA, Black M, Black RE, Brown KH, Campbell H, Carneiro I, Chan KY, Chandramohan D, Chopra M, Cousens S, Darmstadt GL, Meeks Gardner J, Hess SY, Hyder AA, Kapiriri L, Kosek M, Lanata CF, Lansang MA, Lawn J, Tomlinson M, Tsai AC, Webster J (2008). Setting priorities in global child health research investments: guidelines for implementation of CHNRI method. Croat Med J.

[20] Rudan I, El Arifeen S, Bhutta ZA, Black RE, Brooks A, Chan KY, Chopra M, Duke T, Marsh D, Pio A, Simoes EAF, Tamburlini G, Theodoratou E, Weber MW, Whitney CG, Campbell H, Qazi SA (2011). Setting research priorities to reduce global mortality from childhood pneumonia by 2015. PLoS Med.

[21] Tomlinson M, Chopra M, Sanders D, Bradshaw D, Hendricks M, Greenfield D, Black RE, El Arifeen S, Rudan I (2007). Setting priorities in child health research investments for South Africa. PLoS Med.

[22] Viergever RF, Olifson S, Ghaffar A, Terry RF (2010). A checklist for health research priority setting: nine common themes of good practice. Health Res Policy Syst.

[23] NHS Health Research Authority. Defining research. London, UK: National Reserach Ethics Service; 2013.

[24] George A, Young M, Bang A, Chan KY, Rudan I, Victora CG, Chopra M, Rubens C (2011). Setting implementation research priorities to reduce preterm births and stillbirths at the community level. PLoS Med.

[25] Singh H, Giardina TD, Meyer AND, Forjuoh SN, Reis MD, Thomas EJ (2013). Types and origins of diagnostic errors in primary care settings. JAMA Intern Med.

[26] McDonald KM, Matesic B, Contopoulos-Ioannidis DG, Lonhart J, Schmidt E, Pineda N, Ioannidis JPA (2013). Patient safety strategies targeted at diagnostic errors: a systematic review. Ann Intern Med.

[27] Kostopoulou O, Delaney BC, Munro CW (2008). Diagnostic difficulty and error in primary care--a systematic review. Fam Pract.

[28] Lee A, Mills PD, Neily J, Hemphill RR (2014). Root cause analysis of serious adverse events among older patients in the Veterans Health Administration. Jt Comm J Qual Patient Saf.

[29] Marchon SG, Mendes Junior WV (2014). Patient safety in primary health care: a systematic review. Cad Saude Publica.

[30] Callen JL, Westbrook JI, Georgiou A, Li J (2012). Failure to follow-up test results for ambulatory patients: a systematic review. J Gen Intern Med.

[31] Hickner J, Graham DG, Elder NC, Brandt E, Emsermann CB, Dovey S, Phillips R (2008). Testing process errors and their harms and consequences reported from family medicine practices: a study of the American Academy of Family Physicians National Research Network. Qual Saf Health Care.

[32] Hutchinson B (2007). Disparities in healthcare access and use: Yackety-Yack, Yackety-yack. Healthc Policy.

[33] Lasser KE, Himmelstein DU, Woolhandler S (2006). Access to care, health status, and health disparities in the United States and Canada: results of a cross-national population-based survey. Am J Public Health.

[34] Grabovschi C, Loignon C, Fortin M (2013). Mapping the concept of vulnerability related to health care disparities: a scoping review. BMC Health Serv Res.

[35] Gandhi TK, Kachalia A, Thomas EJ, Puopolo AL, Yoon C, Brennan TA, Studdert DM (2006). Missed and delayed diagnoses in the ambulatory setting: a study of closed malpractice claims. Ann Intern Med.

[36] Cook N, Thomson G, Dey P (2014). Managing risk in cancer presentation, detection and referral: a qualitative study of primary care staff views. BMJ Open.

[37] Green T, Atkin K, Macleod U (2015). Cancer detection in primary care: insights from general practitioners. Br J Cancer.

[38] Graber ML, Kissam S, Payne VL, Meyer AND, Sorensen A, Lenfestey N, Tant E, Henriksen K, Labresh K, Singh H (2012). Cognitive interventions to reduce diagnostic error: a narrative review. BMJ Qual Saf.

[39] Akbari A, Mayhew A, Al-Alawi MA, Grimshaw J, Winkens R, Glidewell E, Pritchard C, Thomas R, Fraser C (2008). Interventions to improve outpatient referrals from primary care to secondary care. Cochrane Database Syst Rev.

[40] Hindin MJ, Christiansen S, Ferguson BJ (2013). Setting research priorities for adolescent sexual and reproductive health in low- and middle-income countries. Bull World Health Organ.

[41] Dean S, Rudan I, Althabe F, Webb Girard A, Howson C, Langer A, Lawn J, Reeve ME, Teela KC, Toledano M, Venkatraman CM, Belizan JM, Car J, Chan KY, Chatterjee S, Chitekwe S, Doherty T, Donnay F, Ezzati M, Humayun K, Jack B, Lassi ZS, Martorell R, Poortman Y, Bhutta ZA (2013). Setting research priorities for preconception care in Low- and middle-income countries: aiming to reduce maternal and child mortality and morbidity. PLoS Med.

[42] Lawn JE, Bahl R, Bergstrom S, Bhutta ZA, Darmstadt GL, Ellis M, English M, Kurinczuk JJ, Lee ACC, Merialdi M, Mohamed M, Osrin D, Pattinson R, Paul V, Ramji S, Saugstad OD, Sibley L, Singhal N, Wall SN, Woods D, Wyatt J, Chan KY, Rudan I (2011). Setting research priorities to reduce almost one million deaths from birth asphyxia by 2015. PLoS Med.

[43] Singh H, Thomas EJ, Wilson L, Kelly PA, Pietz K, Elkeeb D, Singhal G (2010). Errors of diagnosis in pediatric practice: a multisite survey. Pediatrics.

[44] Sarkar U, Bonacum D, Strull W, Spitzmueller C, Jin N, López A, Giardina TD, Meyer AND, Singh H (2012). Challenges of making a diagnosis in the outpatient setting: a multi-site survey of primary care physicians. BMJ Qual Saf.

[45] Health Secretary launches new patient safety collaboratives (2014). NHS improving quality.

[46] Richard Grol, Michel Wensing, Martin Eccles et al. Improving patient care: the implementation of change in health care. 2nd Edition. Hoboken, New Jersey, USA: Wiley-Blackwell; 2013.

