# Supplementary material for: Clinician-identified problems and solutions for delayed diagnosis in primary care: a PRIORITIZE study
Source: BMC Fam Pract. 2016 Sep 9;17(1):131. doi: 10.1186/s12875-016-0530-z (PMC5017013; doi:10.1186/s12875-016-0530-z)
Supplement: Additional file 1: — Initial questionnaire on problems and solutions related to medication safety and delayed diagnosis in primary care. (DOCX 65 kb) [file 12875_2016_530_MOESM1_ESM.docx]

**Additional file 1. Initial questionnaire on problems and solutions related to medication safety and delayed diagnosis in primary care**

| 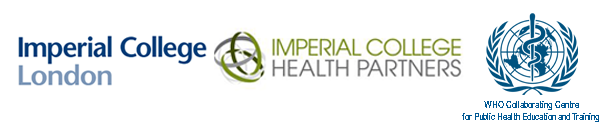 | | | | | | | |
| --- | --- | --- | --- | --- | --- | --- | --- |
| **Patient safety in general practice**      Dear Colleague,    This survey aims to capture (1) your perspective and (2) your solutions for incidents that affect the safety of patients in general practice. It has been designed by the Department of Primary Care and Public Health, Imperial College and Imperial College Health Partners to guide patient safety improvement initiatives in NW London and on a wider scale. The results will be used anonymously. | | | | | | | |
|  | | | | | | | |
| Please specify your role: | | | | | | | |
| Nurse | | Specialist Trainee | Consultant | GP | Social Care Professional | Pharmacist | Other (please specify)  _________________ |
| ○ | | ○ | ○ | ○ | ○ | ○ | ○ |
|  | | | | | | | |
|  | | | | | | | |
| **1. Medication-related problems**  A. Please name **3** main medication-related problems affecting patient safety in general practice. | | | | | | | |
|  |  | | | | | | |
|  | | | | | | | |
| B. How could we reduce or prevent medication-related patient safety incidents in general practice? | | | | | | | |
|  |  | | | | | | |
|  | | | | | | | |
| **2. Delayed Diagnosis**  A. Please name **3** main contributors to delayed diagnosis in general practice. | | | | | | | |
|  |  | | | | | | |
|  | | | | | | | |
| B. What could be done to reduce problems that lead to delayed diagnosis in general practice? | | | | | | | |
|  |  | | | | | | |
|  | | | | | | | |
| **3. Keeping an eye on care**  How could patient safety be better monitored in a GP surgery or a hospital? | | | | | | | |
|  |  | | | | | | |
|  | | | | | | | |
| **4. What's on your mind?**  Kindly give other suggestions for how we could improve patient safety in general practice? | | | | | | | |
|  |  | | | | | | |
|  | | | | | | | |
|  | | | | | | | |
|  | | | | | | | |
| **Thank you very much for your time and effort.**  Your contribution will help improve patient safety in general practice. For more information and to learn more about the outcomes of the study, please contact Dr. Josip Car at [josip.car@imperial.ac.uk](mailto:josip.car@imperial.ac.uk) | | | | | | | |
