# Supplementary material for: Clinician-identified problems and solutions for delayed diagnosis in primary care: a PRIORITIZE study
Source: BMC Fam Pract. 2016 Sep 9;17(1):131. doi: 10.1186/s12875-016-0530-z (PMC5017013; doi:10.1186/s12875-016-0530-z)
Supplement: Additional file 2: — Scoring questionnaire. (DOCX 85 kb) [file 12875_2016_530_MOESM2_ESM.docx]

**Additional file 2. Scoring questionnaire**


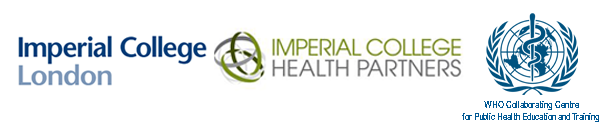


|  |
| --- |

**Patient safety priorities in general practice**

Dear *Colleague,*

Thank you for participating in the first step of this process where you identified patient safety problems in general practice and proposed solutions. They are all listed below. In this second and final step, please indicate your agreement or disagreement with **all** the statements in the table below using the following criteria:

**Y(es) : if you agree**

**N(o) : if you disagree**

**UnS(ure) : if you are aware of the problem, but unsure about the answer**

**UnA(ware) : if you are not sufficiently aware of the problem**

| **Contributors to delayed diagnosis in general practice** | **This patient safety threat is common** | | | | **This patient safety threat leads to high rates of mortality, morbidity and incapacity** | | | | **This patient safety threat affects more lower socio-economic groups or ethnic minorities** | | | | **The consequences of this patient safety threat are costly to the healthcare system** | | | | **This incident is amenable to a solution within 5 years** | | | |
| --- | --- | --- | --- | --- | --- | --- | --- | --- | --- | --- | --- | --- | --- | --- | --- | --- | --- | --- | --- | --- |
|  | Y | N | UnS | UnA | Y | N | UnS | UnA | Y | N | UnS | UnA | Y | N | UnS | UnA | Y | N | UnS | UnA |
| 01. Time constraints such as the 10 minute consultations that lead to incomplete history-taking and patient examination | ○ | ○ | ○ | ○ | ○ | ○ | ○ | ○ | ○ | ○ | ○ | ○ | ○ | ○ | ○ | ○ | ○ | ○ | ○ | ○ |
| 02. A wider scope of GP knowledge is needed e.g. GPs not trained and lack the knowledge, skills and experience in recognizing cancers | ○ | ○ | ○ | ○ | ○ | ○ | ○ | ○ | ○ | ○ | ○ | ○ | ○ | ○ | ○ | ○ | ○ | ○ | ○ | ○ |
| 03. Suboptimal GP knowledge of urgent referral pathways e.g. how and when to use two-weeks cancer referrals | ○ | ○ | ○ | ○ | ○ | ○ | ○ | ○ | ○ | ○ | ○ | ○ | ○ | ○ | ○ | ○ | ○ | ○ | ○ | ○ |
| 04. Not sufficiently considering the diagnosis of cancer at different stages of the decision-making process | ○ | ○ | ○ | ○ | ○ | ○ | ○ | ○ | ○ | ○ | ○ | ○ | ○ | ○ | ○ | ○ | ○ | ○ | ○ | ○ |
| 05. Language and cultural barriers between the GP and the patient | ○ | ○ | ○ | ○ | ○ | ○ | ○ | ○ | ○ | ○ | ○ | ○ | ○ | ○ | ○ | ○ | ○ | ○ | ○ | ○ |
| 06. Reluctance to use scarce resources to investigate unless the condition is critical | ○ | ○ | ○ | ○ | ○ | ○ | ○ | ○ | ○ | ○ | ○ | ○ | ○ | ○ | ○ | ○ | ○ | ○ | ○ | ○ |
| 07. Multiple symptoms or co-morbidities masking the real problem | ○ | ○ | ○ | ○ | ○ | ○ | ○ | ○ | ○ | ○ | ○ | ○ | ○ | ○ | ○ | ○ | ○ | ○ | ○ | ○ |
| 08. NHS cuts, savings and QUIPP leading to GPs' concerns that referral and waiting times will be marked against them | ○ | ○ | ○ | ○ | ○ | ○ | ○ | ○ | ○ | ○ | ○ | ○ | ○ | ○ | ○ | ○ | ○ | ○ | ○ | ○ |
| 09. Lack of continuity of care - seeing different GPs' for the same problem and never being able to follow ‘a case’ through properly | ○ | ○ | ○ | ○ | ○ | ○ | ○ | ○ | ○ | ○ | ○ | ○ | ○ | ○ | ○ | ○ | ○ | ○ | ○ | ○ |
| 10. The rarity of some conditions and undifferentiated or odd presentations | ○ | ○ | ○ | ○ | ○ | ○ | ○ | ○ | ○ | ○ | ○ | ○ | ○ | ○ | ○ | ○ | ○ | ○ | ○ | ○ |
| 11. Inverse care law i.e. those who most need medical care are least likely to receive it. Conversely, those with least need of health care tend to use health services more (and more effectively) | ○ | ○ | ○ | ○ | ○ | ○ | ○ | ○ | ○ | ○ | ○ | ○ | ○ | ○ | ○ | ○ | ○ | ○ | ○ | ○ |
| 12. Psychiatric co-morbidity (the co-occurrence of two or more psychiatric diagnoses) leading doctors to insufficient attention to physical symptoms | ○ | ○ | ○ | ○ | ○ | ○ | ○ | ○ | ○ | ○ | ○ | ○ | ○ | ○ | ○ | ○ | ○ | ○ | ○ | ○ |
| 13. Complicated or unclear CCG pathways | ○ | ○ | ○ | ○ | ○ | ○ | ○ | ○ | ○ | ○ | ○ | ○ | ○ | ○ | ○ | ○ | ○ | ○ | ○ | ○ |
| 14. GP not following the guidelines | ○ | ○ | ○ | ○ | ○ | ○ | ○ | ○ | ○ | ○ | ○ | ○ | ○ | ○ | ○ | ○ | ○ | ○ | ○ | ○ |
| 15. GP adhering too rigidly to the guidelines | ○ | ○ | ○ | ○ | ○ | ○ | ○ | ○ | ○ | ○ | ○ | ○ | ○ | ○ | ○ | ○ | ○ | ○ | ○ | ○ |
| 16. Suboptimal referral pathways to secondary care and a complicated referral process – “referral facilitator” presenting an additional barrier | ○ | ○ | ○ | ○ | ○ | ○ | ○ | ○ | ○ | ○ | ○ | ○ | ○ | ○ | ○ | ○ | ○ | ○ | ○ | ○ |
| 17. Jumping to a conclusion about the diagnosis too early during the consultation | ○ | ○ | ○ | ○ | ○ | ○ | ○ | ○ | ○ | ○ | ○ | ○ | ○ | ○ | ○ | ○ | ○ | ○ | ○ | ○ |
| 18. Constant pressure on GPs (in their “gatekeeper” role) to decrease referral rates and admissions | ○ | ○ | ○ | ○ | ○ | ○ | ○ | ○ | ○ | ○ | ○ | ○ | ○ | ○ | ○ | ○ | ○ | ○ | ○ | ○ |
| 19. Pressure not to refer unless ‘red flags’ for 2-week wait | ○ | ○ | ○ | ○ | ○ | ○ | ○ | ○ | ○ | ○ | ○ | ○ | ○ | ○ | ○ | ○ | ○ | ○ | ○ | ○ |
| 20. Patient’s delay in presenting symptoms (e.g. “I have had blood in my urine for a year”) | ○ | ○ | ○ | ○ | ○ | ○ | ○ | ○ | ○ | ○ | ○ | ○ | ○ | ○ | ○ | ○ | ○ | ○ | ○ | ○ |
| 21. Lack of patient awareness of ‘red flag’ symptoms | ○ | ○ | ○ | ○ | ○ | ○ | ○ | ○ | ○ | ○ | ○ | ○ | ○ | ○ | ○ | ○ | ○ | ○ | ○ | ○ |
| 22. Patients attending other services such as A&E, walk-in centers instead of seeing their own GP | ○ | ○ | ○ | ○ | ○ | ○ | ○ | ○ | ○ | ○ | ○ | ○ | ○ | ○ | ○ | ○ | ○ | ○ | ○ | ○ |
| 23. Reports of investigations sent to GP without clear conclusions and suggestions for referral. Hence the GP ends up interpreting how soon the referrals should be made | ○ | ○ | ○ | ○ | ○ | ○ | ○ | ○ | ○ | ○ | ○ | ○ | ○ | ○ | ○ | ○ | ○ | ○ | ○ | ○ |
| 24. Difficult and delayed access to diagnostics or expertise when patients do not fit or match the 2 week wait criteria | ○ | ○ | ○ | ○ | ○ | ○ | ○ | ○ | ○ | ○ | ○ | ○ | ○ | ○ | ○ | ○ | ○ | ○ | ○ | ○ |
| 25. Long waiting times and lack of open access to diagnostic tests, investigations and their results e.g. CT and MRI | ○ | ○ | ○ | ○ | ○ | ○ | ○ | ○ | ○ | ○ | ○ | ○ | ○ | ○ | ○ | ○ | ○ | ○ | ○ | ○ |
| 26. Difficult access to a GP: appointment systems are complicated and must seem impenetrable to elderly and vulnerable patients. Also, lack of suitable time appointments for the working population | ○ | ○ | ○ | ○ | ○ | ○ | ○ | ○ | ○ | ○ | ○ | ○ | ○ | ○ | ○ | ○ | ○ | ○ | ○ | ○ |
| 27. Poor communication between secondary and primary care; e.g. investigations that are ordered by secondary care are not visible in primary care | ○ | ○ | ○ | ○ | ○ | ○ | ○ | ○ | ○ | ○ | ○ | ○ | ○ | ○ | ○ | ○ | ○ | ○ | ○ | ○ |
| 28. Urgent 2 week waits are often delayed by secondary care while waiting for investigations to be carried out | ○ | ○ | ○ | ○ | ○ | ○ | ○ | ○ | ○ | ○ | ○ | ○ | ○ | ○ | ○ | ○ | ○ | ○ | ○ | ○ |
| 29. GP not taking action when abnormal results of investigations occur | ○ | ○ | ○ | ○ | ○ | ○ | ○ | ○ | ○ | ○ | ○ | ○ | ○ | ○ | ○ | ○ | ○ | ○ | ○ | ○ |
| 30. Lack of follow up and “safety netting” of initial symptoms | ○ | ○ | ○ | ○ | ○ | ○ | ○ | ○ | ○ | ○ | ○ | ○ | ○ | ○ | ○ | ○ | ○ | ○ | ○ | ○ |
| 31. Poor local imaging and reporting | ○ | ○ | ○ | ○ | ○ | ○ | ○ | ○ | ○ | ○ | ○ | ○ | ○ | ○ | ○ | ○ | ○ | ○ | ○ | ○ |
| 32. The involvement of a number of different specialists in the same case can result in each assuming the other will follow-up | ○ | ○ | ○ | ○ | ○ | ○ | ○ | ○ | ○ | ○ | ○ | ○ | ○ | ○ | ○ | ○ | ○ | ○ | ○ | ○ |
| 33. GPs’ burnout and exhaustion | ○ | ○ | ○ | ○ | ○ | ○ | ○ | ○ | ○ | ○ | ○ | ○ | ○ | ○ | ○ | ○ | ○ | ○ | ○ | ○ |
| **Please add any further ideas or comments that you believe are important and should be included in this study.** | | | | | | | | | | | | | | | | | | | | |
|  | |  | | | | | | | | | | | | | | | | | | |

| **Y(es) : if you agree**  **N(o) : if you disagree**  **UnS(ure) : if you are aware of the problem, but unsure about the answer**  **UnA(ware) : if you are not sufficiently aware of the problem** | | | | | | | | | | | | | |
| --- | --- | --- | --- | --- | --- | --- | --- | --- | --- | --- | --- | --- | --- |
| **Suggested solutions to problems that lead to delayed diagnosis in general practice** | | **This solution is cost-effective** | | | | **The implementation of this solution is feasible** | | | | **This solution would save lives** | | | |
|  |  | Y | N | UnS | UnA | Y | N | UnS | UnA | Y | N | UnS | UnA |
| 01. Better training of GPs in” spotting warning signs of serious conditions”, diagnosis that are easily missed” and “safety netting” | | ○ | ○ | ○ | ○ | ○ | ○ | ○ | ○ | ○ | ○ | ○ | ○ |
| 02. Training in decision making and reinforcing the concept on ongoing reflection to continuous consideration of differential diagnosis | | ○ | ○ | ○ | ○ | ○ | ○ | ○ | ○ | ○ | ○ | ○ | ○ |
| 03. More training to be available for GP Practice staff at all levels | | ○ | ○ | ○ | ○ | ○ | ○ | ○ | ○ | ○ | ○ | ○ | ○ |
| 04. To have “affordable” GP update courses | | ○ | ○ | ○ | ○ | ○ | ○ | ○ | ○ | ○ | ○ | ○ | ○ |
| 05. Mandatory postgraduate training for GPs on “early signs of cancer” | | ○ | ○ | ○ | ○ | ○ | ○ | ○ | ○ | ○ | ○ | ○ | ○ |
| 06. Publishing information about common symptoms | | ○ | ○ | ○ | ○ | ○ | ○ | ○ | ○ | ○ | ○ | ○ | ○ |
| 07. Improving GPs’ listening skills (‘the patient is telling you the diagnosis’) | | ○ | ○ | ○ | ○ | ○ | ○ | ○ | ○ | ○ | ○ | ○ | ○ |
| 08. Examining patients more often (i.e. 'if you don't put your finger in it, you’ll put your foot in it') | | ○ | ○ | ○ | ○ | ○ | ○ | ○ | ○ | ○ | ○ | ○ | ○ |
| 09. Improve note keeping | | ○ | ○ | ○ | ○ | ○ | ○ | ○ | ○ | ○ | ○ | ○ | ○ |
| 10. Improve handovers | | ○ | ○ | ○ | ○ | ○ | ○ | ○ | ○ | ○ | ○ | ○ | ○ |
| 11. More political honesty about what the NHS can do and cannot do | | ○ | ○ | ○ | ○ | ○ | ○ | ○ | ○ | ○ | ○ | ○ | ○ |
| 12. Have easier access to secondary care for the patients that GPs are worried about | | ○ | ○ | ○ | ○ | ○ | ○ | ○ | ○ | ○ | ○ | ○ | ○ |
| 13. The system to encourage and try to make sure the patient sees the same doctor who knows them best e.g. especially for test results etc. | | ○ | ○ | ○ | ○ | ○ | ○ | ○ | ○ | ○ | ○ | ○ | ○ |
| 14. Regular reviews of how much guidelines are being followed | | ○ | ○ | ○ | ○ | ○ | ○ | ○ | ○ | ○ | ○ | ○ | ○ |
| 15. Review of every delayed diagnosis to learn how, why and whether it could be prevented in the future | | ○ | ○ | ○ | ○ | ○ | ○ | ○ | ○ | ○ | ○ | ○ | ○ |
| 16. Improving GPs' awareness of how pressured they are feeling when running late | | ○ | ○ | ○ | ○ | ○ | ○ | ○ | ○ | ○ | ○ | ○ | ○ |
| 17. Less fragmentation on disease specific pathways | | ○ | ○ | ○ | ○ | ○ | ○ | ○ | ○ | ○ | ○ | ○ | ○ |
| 18. Not to penalize referrals despite them not fitting guidelines | | ○ | ○ | ○ | ○ | ○ | ○ | ○ | ○ | ○ | ○ | ○ | ○ |
| 19. Public health “patient awareness and education” campaigns to encourage patients to present early e.g. FAST or cough for more than 3 weeks | | ○ | ○ | ○ | ○ | ○ | ○ | ○ | ○ | ○ | ○ | ○ | ○ |
| 20. Better ways of informing patients that their results are ready and what the next best steps would be | | ○ | ○ | ○ | ○ | ○ | ○ | ○ | ○ | ○ | ○ | ○ | ○ |
| 21. Educate patients to keep appointments | | ○ | ○ | ○ | ○ | ○ | ○ | ○ | ○ | ○ | ○ | ○ | ○ |
| 22. To have more rigorous systems in place for communicating abnormal results to patients | | ○ | ○ | ○ | ○ | ○ | ○ | ○ | ○ | ○ | ○ | ○ | ○ |
| 23. Clear referral guidelines and pathways for other common conditions (not just cancer) | | ○ | ○ | ○ | ○ | ○ | ○ | ○ | ○ | ○ | ○ | ○ | ○ |
| 24. Greater use of decision support software (e.g. the Macmillan add-in that flags up concerning cancer symptoms even if it has spread across several consultations) | | ○ | ○ | ○ | ○ | ○ | ○ | ○ | ○ | ○ | ○ | ○ | ○ |
| 25. Hospitals to follow up on patients who miss their appointments (currently it is unclear who is responsible for this follow up) | | ○ | ○ | ○ | ○ | ○ | ○ | ○ | ○ | ○ | ○ | ○ | ○ |
| 26. Direct hotlines to specialists to discuss patient problems | | ○ | ○ | ○ | ○ | ○ | ○ | ○ | ○ | ○ | ○ | ○ | ○ |
| 27. Longer GP opening hours | | ○ | ○ | ○ | ○ | ○ | ○ | ○ | ○ | ○ | ○ | ○ | ○ |
| **Please add any further ideas or comments that you believe are important and should be included in this study.** | | | | | | | | | | | | | |
|  |  | | | | | | | | | | | | |
| **Is there anything else for patient safety in general practice that you would like to share with us?** | | | | | | | | | | | | | |
|  |  | | | | | | | | | | | | |
| **Thank you very much for your time and effort.**  Your contribution will help improve patient safety in general practice.  For more information and to learn more about the outcomes of the study, please contact Dr. Josip Car at [josip.car@imperial.ac.uk](mailto:josip.car@imperial.ac.uk) | | | | | | | | | | | | | |
