# Supplementary material for: Clinician-identified problems and solutions for delayed diagnosis in primary care: a PRIORITIZE study
Source: BMC Fam Pract. 2016 Sep 9;17(1):131. doi: 10.1186/s12875-016-0530-z (PMC5017013; doi:10.1186/s12875-016-0530-z)
Supplement: Additional file 3: — Categories of Organizational Interventions to Decrease Diagnostic Errors. (DOCX 13 kb) [file 12875_2016_530_MOESM3_ESM.docx]

**Additional file 3. Categories of Organizational Interventions to Decrease Diagnostic Errors***

- Technique: Changes in equipment, procedures, and clinical approaches used in clinical practice
- Personnel changes: Introduction of additional or replacement of healthcare providers
- Educational interventions: Educational strategies, training etc.
- Structured process changes: Improvements to the existing or implementation of additional stages or process in the diagnostic pathway
- Technology-based system interventions: Implementation at the system level of tools, such as computer assistive diagnostic aids, decision-support algorithms, text message alerting, and pager alerts
- Additional review methods: Introduction of additional independent reviews in the diagnostic pathway*

(*from McDonald KM1, Matesic B, Contopoulos-Ioannidis DG, Lonhart J, Schmidt E, Pineda N, Ioannidis JP. Patient safety strategies targeted at diagnostic errors: a systematic review. Ann Intern Med;158(5 Pt 2):381-9.)
