# Supplementary material for: Clinician-identified problems and solutions for delayed diagnosis in primary care: a PRIORITIZE study
Source: BMC Fam Pract. 2016 Sep 9;17(1):131. doi: 10.1186/s12875-016-0530-z (PMC5017013; doi:10.1186/s12875-016-0530-z)
Supplement: Additional file 4: — Characteristics of the respondents to the initial questionnaire. (DOCX 12 kb) [file 12875_2016_530_MOESM4_ESM.docx]

**Additional file 4. Characteristics of the respondents to the initial questionnaire**

- Total number of survey respondents: 119
- GPs: 89, 75%
- GP Trainees: 19, 16%
- Foundation doctors: 4, 3%
- Blank: 3, 2%
- Nurses: 1, 0.8%
- Specialist trainees in Public Health: 1, 0.8%
- Consultants: 1, 0.8%
- Pharmacists: 1, 0.8%
