# Supplementary material for: Clinician-identified problems and solutions for delayed diagnosis in primary care: a PRIORITIZE study
Source: BMC Fam Pract. 2016 Sep 9;17(1):131. doi: 10.1186/s12875-016-0530-z (PMC5017013; doi:10.1186/s12875-016-0530-z)
Supplement: Additional file 5: — Ranking of all (33) problems leading to delayed diagnosis from primary care clinicians’ perspective (AEA range: 0 to 1). (DOCX 19 kb) [file 12875_2016_530_MOESM5_ESM.docx]

**Additional file 5. Ranking of all (33) problems leading to delayed diagnosis from primary care clinicians’ perspective (AEA range: 0 to 1)**

| **Highlighted delayed diagnosis related problems in primary care** | **Type of factor contributing to delayed diagnosis** | **Breakdown points in the Diagnostic Process** | **TPS** | **AEA** | **Responsiveness to solution** | **Frequency** | **Economic impact** | **Severity** | **Inequity** |
| --- | --- | --- | --- | --- | --- | --- | --- | --- | --- |
| *Poor communication between secondary and primary care; e.g. investigations that are ordered by secondary care are not visible in primary care* | System factor | Referral & consultation | **1** | **0,78** | **1** | **1** | **2** | **3** | **25** |
| *Inverse care law i.e. those who most need medical care are least likely to receive it. Conversely, those with least need of health care tend to use health services more (and more effectively)* | System and Patient-related factor | Access & presentation | **2** | **0,74** | **25** | **4** | **1** | **1** | **5** |
| *Patients attending other services such as A&E, walk-in centres instead of seeing their own GP* | System and Patient-related factor | Access & presentation | **3** | **0,69** | **9** | **7** | **4** | **13** | **4** |
| *Multiple symptoms or co-morbidities masking the real problem* | Cognitive factor | Patient-practitioner encounter | **4** | **0,71** | **27** | **2** | **3** | **2** | **7** |
| *Lack of continuity of care - seeing different GPs' for the same problem and never being able to follow ‘a case’ through properly* | System factor | Patient-practitioner encounter | **5** | **0,69** | **10** | **6** | **5** | **4** | **9** |
| *Time constraints such as the 10 minute consultations that lead to incomplete history-taking and patient examination* | System factor | Patient-practitioner encounter | **6** | **0,68** | **6** | **3** | **9** | **9** | **8** |
| *Lack of patient awareness of ‘red flag’ symptoms* | Patient factor | Access & presentation | **7** | **0,69** | **18** | **10** | **8** | **7** | **3** |
| *Patient’s delay in presenting symptoms (e.g. “I have had blood in my urine for a year”)* | Patient factor | Access & presentation | **8** | **0,68** | **21** | **14** | **7** | **6** | **2** |
| *Psychiatric co-morbidity (the co-occurrence of two or more psychiatric diagnoses) leading doctors to insufficient attention to physical symptoms* | Cognitive factor | Patient-practitioner encounter | **9** | **0,68** | **20** | **9** | **6** | **5** | **6** |
| *Language and cultural barriers between the GP and the patient* | System and Patient-related factor | Patient-practitioner encounter | **10** | **0,64** | **22** | **13** | **11** | **10** | **1** |
| *GPs’ burnout and exhaustion* | System factor | - | **11** | **0,61** | **15** | **5** | **10** | **8** | **15** |
| *Difficult and delayed access to diagnostics or expertise when patients do not fit or match the 2 week wait criteria* | System factor | Diagnostic testing | **12** | **0,63** | **3** | **12** | **12** | **11** | **16** |
| *Reports of investigations sent to GP without clear conclusions and suggestions for referral. Hence the GP ends up interpreting how soon the referrals should be made* | System factor | Referral & consultation | **13** | **0,65** | **2** | **15** | **14** | **14** | **29** |
| *Constant pressure on GPs (in their “gatekeeper” role) to decrease referral rates and admissions* | System factor | Referral & consultation | **14** | **0,54** | **12** | **8** | **18** | **16** | **14** |
| *Long waiting times and lack of open access to diagnostic tests, investigations and their results e.g. CT and MRI* | System factor | Diagnostic testing | **15** | **0,60** | **4** | **17** | **15** | **15** | **21** |
| *NHS cuts, savings and QUIPP leading to GPs' concerns that referral and waiting times will be marked against them* | System factor | Referral & consultation | **16** | **0,54** | **11** | **11** | **13** | **12** | **20** |
| *Difficult access to a GP: appointment systems are complicated and must seem impenetrable to elderly and vulnerable patients. Also, lack of suitable time appointments for the working population* | System factor | Access & Presentation | **17** | **0,51** | **13** | **19** | **16** | **19** | **10** |
| *Pressure not to refer unless ‘red flags’ for 2 week wait* | System factor | Referral & Consultation | **18** | **0,56** | **8** | **16** | **17** | **17** | **18** |
| *Suboptimal referral pathways to secondary care and a complicated referral process – “referral facilitator” presenting an additional barrier* | System factor | Referral & Consultation | **19** | **0,51** | **5** | **18** | **19** | **23** | **23** |
| *The involvement of a number of different specialists in the same case can result in each assuming the other will follow-up* | System factor | Follow-up | **20** | **0,50** | **7** | **22** | **20** | **21** | **19** |
| *The rarity of some conditions and undifferentiated or odd presentations* | Cognitive factor | Patient-practitioner encounter | **21** | **0,49** | **32** | **20** | **21** | **18** | **12** |
| *Lack of follow up and “safety netting” of initial symptoms* | System factor | Follow-up | **22** | **0,48** | **24** | **25** | **24** | **20** | **11** |
| *Jumping to a conclusion about the diagnosis too early during the consultation* | Cognitive factor | Patient-practitioner encounter | **23** | **0,42** | **29** | **23** | **23** | **22** | **13** |
| *Complicated or unclear CCG pathways* | System factor | Referral & Consultation | **24** | **0,45** | **17** | **21** | **22** | **26** | **27** |
| *Urgent 2 week waits are often delayed by secondary care while waiting for investigations to be carried out* | System factor | Referral & Consultation | **25** | **0,43** | **14** | **26** | **28** | **24** | **26** |
| *Not sufficiently considering the diagnosis of cancer at different stages of the decision-making process* | Cognitive factor | Patient-practitioner encounter | **26** | **0,45** | **19** | **28** | **25** | **25** | **22** |
| *Reluctance to use scarce resources to investigate unless the condition is critical* | System factor | Diagnostic testing | **27** | **0,44** | **23** | **27** | **26** | **27** | **24** |
| *Poor local imaging and reporting* | System factor | Diagnostic testing | **28** | **0,45** | **16** | **29** | **30** | **30** | **31** |
| *GP not taking action when abnormal results of investigations occur* | Cognitive factor | Follow-up | **29** | **0,47** | **28** | **31** | **29** | **28** | **28** |
| *A wider scope of GP knowledge is needed e.g. GPs not trained and lack the knowledge, skills and experience in recognizing cancers* | Cognitive factor | Patient-practitioner encounter | **30** | **0,48** | **30** | **32** | **27** | **29** | **17** |
| *GP adhering too rigidly to the guidelines* | Cognitive factor | Patient-practitioner encounter | **31** | **0,42** | **31** | **24** | **31** | **31** | **30** |
| *GP not following the guidelines* | Cognitive factor | Patient-practitioner encounter | **32** | **0,45** | **33** | **30** | **33** | **32** | **32** |
| *Suboptimal GP knowledge of urgent referral pathways e.g. how and when to use two-weeks cancer referrals* | Cognitive factor | Referral & consultation | **33** | **0,58** | **26** | **33** | **32** | **33** | **33** |

***AEA –average expert agreement; TPS – total priority score***
