# Supplementary material for: Clinician-identified problems and solutions for delayed diagnosis in primary care: a PRIORITIZE study
Source: BMC Fam Pract. 2016 Sep 9;17(1):131. doi: 10.1186/s12875-016-0530-z (PMC5017013; doi:10.1186/s12875-016-0530-z)
Supplement: Additional file 6: — Ranking of all (27) solutions to delayed diagnosis from primary care clinicians’ perspective (AEA range: 0 to 1). (DOCX 17 kb) [file 12875_2016_530_MOESM6_ESM.docx]

**Additional file 6. Ranking of all (27) solutions to delayed diagnosis from primary care clinicians’ perspective (AEA range: 0 to 1)**

| **Proposed solution for delayed diagnosis problems in primary care** | **Categories of Organizational Interventions to Decrease Diagnostic Errors** | **Breakdown points in the Diagnostic Process** | **TPS** | **AEA** | **Cost-effectiveness** | **Feasibility** | **Saving Lives** |
| --- | --- | --- | --- | --- | --- | --- | --- |
| *To have more rigorous systems in place for communicating abnormal results to patients* | Structured-process change | Follow-up | **1** | **0,85** | **4** | **1** | **1** |
| *Direct hotlines to specialists to discuss patient problems* | Structured-process change | Referral & consultation | **2** | **0,86** | **1** | **3** | **7** |
| *Clear referral guidelines and pathways for other common conditions (not just cancer)* | Structured-process change | Referral & consultation | **3** | **0,82** | **2** | **2** | **12** |
| *Improve handovers* | Structured-process change | Referral & consultation | **4** | **0,81** | **5** | **14** | **5** |
| *To have “affordable” GP update courses* | Educational | Patient-practitioner encounter | **5** | **0,79** | **3** | **4** | **14** |
| *Better training of GPs in ”spotting warning signs of serious conditions”, diagnosis that are easily missed” and “safety netting”* | Educational | Patient-practitioner encounter | **6** | **0,78** | **8** | **7** | **6** |
| *Review of every delayed diagnosis to learn how, why and whether it could be prevented in the future* | Additional review & education | - | **7** | **0,77** | **12** | **13** | **3** |
| *Better ways of informing patients that their results are ready and what the next best steps would be* | Structured-process change | Follow-up | **8** | **0,76** | **11** | **6** | **9** |
| *Training in decision making and reinforcing the concept on ongoing reflection to continuous consideration of differential diagnosis* | Educational | Patient-practitioner encounter | **9** | **0,77** | **10** | **8** | **8** |
| *Have easier access to secondary care for the patients that GPs are worried about* | Structured-process change | Referral & consultation | **10** | **0,77** | **14** | **12** | **4** |
| *Improve note keeping* | Technique | Patient-practitioner encounter | **11** | **0,76** | **9** | **15** | **10** |
| *The system to encourage and try to make sure the patient sees the same doctor who knows them best e.g. especially for test results etc.* | Structured-process change | Patient-practitioner encounter | **12** | **0,75** | **13** | **18** | **2** |
| *Publicising information about common symptoms* | Educational | Access & Presentation | **13** | **0,73** | **17** | **9** | **11** |
| *Not to penalize referrals despite them not fitting guidelines* | Structured-process change | Referral & consultation | **14** | **0,67** | **18** | **5** | **13** |
| *Hospitals to follow up on patients who miss their appointments (currently it is unclear who is responsible for this follow up)* | Structured-process change | Access & Presentation | **15** | **0,68** | **16** | **10** | **15** |
| *More training to be available for GP Practice staff at all levels* | Educational | Patient-practitioner encounter | **16** | **0,63** | **15** | **17** | **19** |
| *More political honesty about what the NHS can do and cannot do* | Structured-process change | - | **17** | **0,65** | **6** | **11** | **25** |
| *Public health “patient awareness and education” campaigns to encourage patients to present early e.g. FAST or cough for more than 3 weeks* | Patient intervention | Access & Presentation | **18** | **0,64** | **20** | **16** | **18** |
| *Educate patients to keep appointments* | Patient intervention | Access & Presentation | **19** | **0,64** | **7** | **21** | **23** |
| *Greater use of decision support software (e.g. the Macmillan add-in that flags up concerning cancer symptoms even if it has spread across several consultations)* | Technology-based system intervention | Patient-practitioner encounter | **20** | **0,56** | **22** | **19** | **17** |
| *Examining patients more often (i.e. 'if you don't put your finger in it, you’ll put your foot in it')* | Structured-process change | Patient-practitioner encounter | **21** | **0,58** | **19** | **24** | **16** |
| *Less fragmentation on disease specific pathways* | Structured-process change | Referral & consultation | **22** | **0,54** | **21** | **23** | **20** |
| *Improving GPs’ listening skills (‘the patient is telling you the diagnosis’)* | Educational | Patient-practitioner encounter | **23** | **0,54** | **24** | **22** | **21** |
| *Mandatory postgraduate training for GPs on “early signs of cancer”* | Educational | Patient-practitioner encounter | **24** | **0,52** | **25** | **25** | **22** |
| *Improving GPs' awareness of how pressured they are feeling when running late* | Educational | Patient-practitioner encounter | **25** | **0,51** | **23** | **20** | **24** |
| *Regular reviews of how much guidelines are being followed* | Additional review | Patient-practitioner encounter | **26** | **0,42** | **26** | **26** | **26** |
| *Longer GP opening hours* | Structure-process change | Access & Presentation | **27** | **0,55** | **27** | **27** | **27** |

***AEA –average expert agreement; TPS – total priority score***
